# Niedrig dosierte Ganzhaut‐Elektronenbestrahlung bei erythrodermatischer Mycosis fungoides und Sézary‐Syndrom: Ergebnisse aus der prospektiven S‐MISR‐Studie

**DOI:** 10.1111/ddg.15851_g

**Published:** 2025-12-11

**Authors:** Khaled Elsayad, Christian Kandler, Jan Jakob Siats, Niklas Pepper, Moritz Fabian Danzer, Gabor Dobos, Elisabeth Livingstone, Susanne Melchers, Johannes Kleemann, Julia Hyun, Claudia Pföhler, Peter von den Driesch, Jan P. Nicolay, Rudolf Stadler, Hans Theodor Eich

**Affiliations:** ^1^ Klinik für Strahlentherapie – Radioonkologie Universitätsklinikum Münster; ^2^ Institut für Biometrie und Klinische Forschung Universität Münster; ^3^ Klinik für Dermatologie Venerologie und Allergologie Charité – Universitätsmedizin Berlin Gemeinsame Medizinische Fakultät der Freien Universität Berlin und der Humboldt‐Universität Berlin; ^4^ Klinik für Dermatologie Universitätsklinikum Essen; ^5^ Klinik für Dermatologie Venerologie und Allergologie Universitätsklinikum Mannheim; ^6^ Klinik für Dermatologie Venerologie und Allergologie Goethe‐Universität Frankfurt am Main; ^7^ Klinik für Dermatologie Venerologie und Allergologie Helios St. Johannes Krankenhaus Duisburg; ^8^ Klinik für Dermatologie Venerologie und Allergologie Universitätsklinikum des Saarlandes Homburg/Saar; ^9^ Zentrum für Dermatologie Phlebologie und Allergologie | Klinikum Stuttgart Bad Cannstatt Stuttgart Deutschland; ^10^ Universitätsklinik für Dermatologie Venerologie Allergologie und Phlebologie Hautkrebszentrum Johannes Wesling Klinikum UKRUB Universität Bochum, Minden

**Keywords:** Blutbeteiligung, gesundheitsbezogene Lebensqualität, Immuntherapie, Leukämietyp, Pruritus, Strahlentherapie, S‐MISR‐Register, blood involvement, health‐related quality of life, immunotherapy, leukemic type, pruritus, Radiotherapy, S‐MISR registry

## Abstract

**Hintergrund:**

Bei der erythrodermatischen Mycosis fungoides (eMF) und dem Sézary‐Syndrom (SS) sind systemische Therapien häufig mit verzögerten und nicht anhaltenden klinischen Remissionen verbunden.

**Patienten und Methodik:**

35 Patienten mit eMF und SS wurden mittels Ganzhautelektronenbestrahlung (*total skin electron beam therapy*, TSEBT) behandelt. Analysiert wurden Ansprechraten, patientenberichtete Verläufe, Überlebensraten und die mediane Zeit bis zur nächsten Behandlung (*time to next treatment*, TTNT) und Progressionsfreies Überleben (*progression‐free survival*, PFS).

**Ergebnisse:**

In die Studie wurden 21 Patienten mit SS und 14 Patienten mit eMF eingeschlossen. Die applizierte Strahlendosis betrug im Median 12 Gy. Nach der TSEBT erhielten 25 Patienten (71%) eine weitere Therapie. Die Gesamtansprechrate nach 3 Monaten lag bei 89%. Die mediane TTNT lag bei 20 Monaten, das mediane PFS bei 14 Monaten. Bei den Patienten zeigten sich eine deutliche Verringerung des Juckreizes, eine Abnahme der Hautläsionen und eine Verbesserung der Lebensqualität. Die mediane TTNT und das PFS waren in der Gruppe mit Anschlusstherapie nach TSEBT länger als bei Patienten ohne weitere Therapie. Die Rate von Strahlentherapie‐Toxizitäten ≥ Grad 3 betrug 6%. Aus translationaler Perspektive konnten mehrere potenzielle Biomarker im peripheren Blut identifiziert werden.

**Schlussfolgerungen:**

Patienten mit eMF und SS können wirksam und sicher mit niedrig dosierten TSEBT behandelt werden, sodass anschließende Therapien sowie weitere Behandlungen möglich sind. Die Patienten zeigten eine deutliche Verbesserung der Lebensqualität bei gleichzeitig niedriger Toxizität.

## EINLEITUNG

Das Sézary‐Syndrom (SS) ist ein seltenes primär kutanes T‐Zell‐Lymphom mit einer jährlichen Inzidenz von < 0,2 pro 1 Million Menschen.^1^, ^2^ Neben der Erythrodermie ist das SS durch zirkulierende Tumorzellen und eine erhöhte Tumorlast im Blut (B2) mit aberranten T‐Zell‐Populationen gekennzeichnet (CD4^+^/CD26^−^‐ oder CD4^+^/CD7^−^‐Zellen, nachweisbar mittels Durchflusszytometrie).^3^ Ein kleiner Teil der MF‐Patienten (< 5%) weist eine diffuse Erythrodermie (T4) mit geringer Tumorlast im Blut (B0–B1) auf.^4^ Die Prognose des SS ist ungünstiger als die der MF.^5^ Die prognostische Differenz zwischen eMF und SS ist bislang nicht abschließend charakterisiert.^3^ Klinisch leiden die Patienten in der Regel an mäßigen bis schweren Hauterscheinungen, welche die Lebensqualität (*quality of life*, QoL) stark beeinträchtigen.^6^ Die meisten SS‐Patienten werden *de novo* diagnostiziert; ein kleiner Teil der Fälle kann jedoch sekundär zur MF auftreten.^7^ Die Behandlungen für Patienten mit eMF und SS unterscheiden sich von Zentrum zu Zentrum und haben in der Regel einen verzögerten und nicht dauerhaften klinischen Nutzen für die Patienten.^7^ Für die Behandlung einer eMF können die Empfehlungen für SS berücksichtigt werden.^8^ Die Strahlentherapie (*radiotherapy*, RT) kann zu einer Besserung der klinischen Symptome führen.^9^ Bislang liegen jedoch nur wenige Studien mit guten Ergebnissen zur niedrig dosierten Ganzhautelektronenbestrahlung (*total skin electron beam therapy*, TSEBT) bei Patienten mit eMF und SS vor.^10^ Zusätzlich zeigen Daten, dass eine TSEBT die Tumorlast im peripheren Blut verringern könnte.^11^, ^12^ So deuten einige wenige retrospektive Studien darauf hin, dass lösliche Biomarker mit dem Schweregrad der Erkrankung korrelieren^13^ und die Krankheitsaktivität widerspiegeln könnten.^14^, ^15^

In dieser Studie soll die Wirksamkeit einer niedrig dosierten TSEBT und die Auswirkungen einer Anschlusstherapie bei Patienten mit eMF und SS untersucht werden. Darüber hinaus werden potenzielle Biomarker im peripheren Blut vom Ausgangswert bis zur Nachuntersuchung analysiert.

## MATERIAL UND METHODIK

### Studiendesign

**ABBILDUNG 1:**
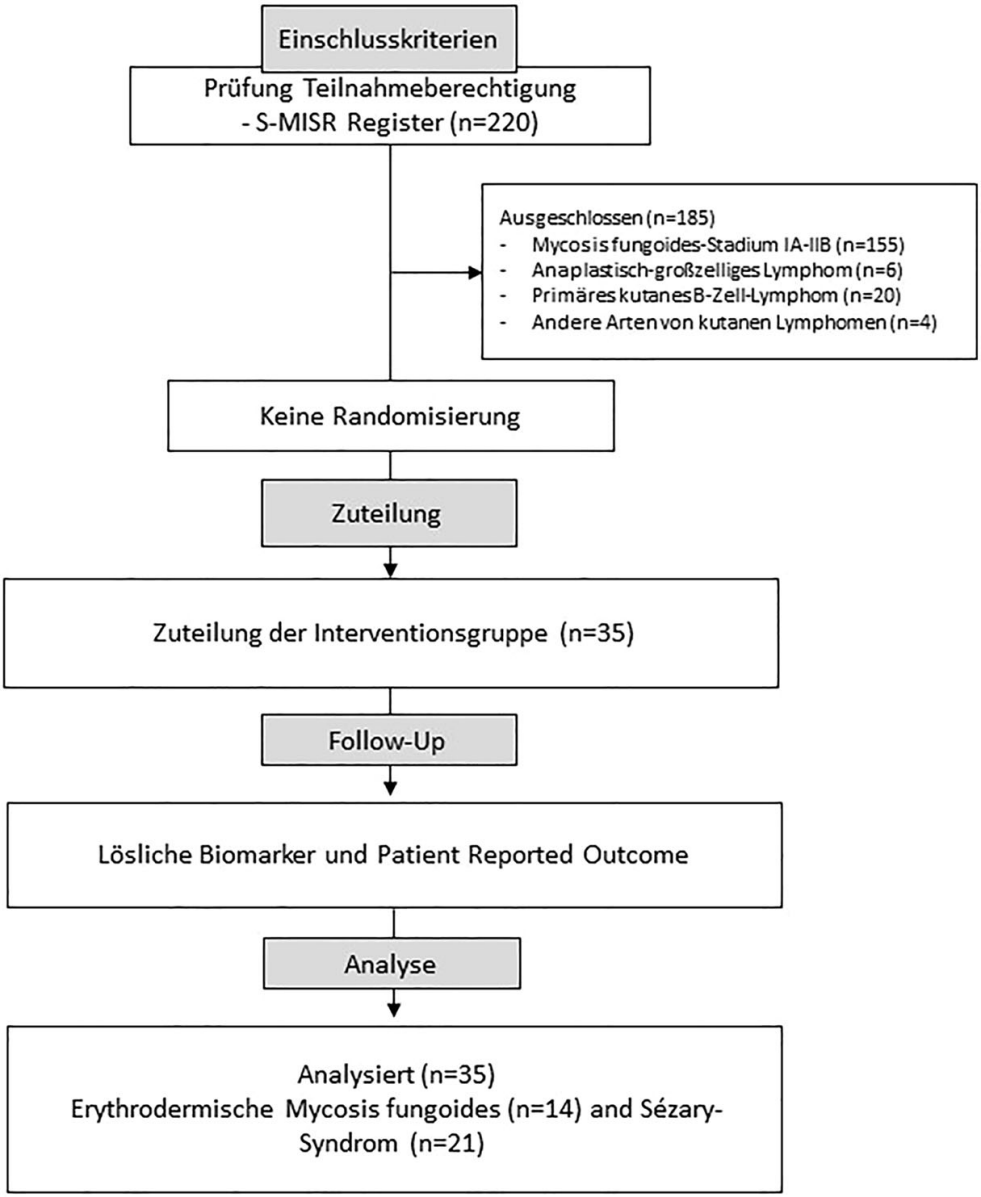
CONSORT‐Flowchart für Patienten mit erythrodermatischer Mycosis fungoides und Sézary‐Syndrom, die eine niedrig dosierte Ganzhautelektronenbestrahlung erhalten (n = 35).

Bei der vorliegenden Studie handelt es sich um eine prospektive Beobachtungsstudie von Patienten mit eMF und SS. Eingeschlossen wurden Patienten, die von angeschlossenen dermatologischen Zentren in Deutschland überwiesen wurden (TrialSearch (WHO) und Registernummer für das Deutsche Register Klinischer Studien: DRKS00030375). Patienten mit klinisch und histologisch bestätigter aktiver eMF (Stadium IIIA–IIIB) und SS (Stadium IVA) wurden in die Analyse einbezogen (Abbildung 1).

### Patienten

Die Einschlusskriterien waren eine histologisch bestätigte Diagnose von eMF und SS, ein unzureichendes Ansprechen auf mindestens eine vorangegangene Therapie, ein Alter von ≥ 18 Jahren, keine stattgehabte systemische Therapie innerhalb der letzten 4 Wochen und keine topische Therapie innerhalb der letzten 2 Wochen sowie eine Lebenserwartung von mehr als 6 Monaten. Zu den Ausschlusskriterien gehörten: Eine frühere oder aktive bösartige Hauterkrankung, eine frühere oder aktive nicht‐infektiöse Pneumonitis, die Steroide erfordert, eine Lungenfibrose, Schwangerschaft oder Stillzeit und Patienten, die auf eine Stammzelltransplantation warteten. In dieser Studie wurden die nachfolgenden Therapien zur Aufrechterhaltung der Remission innerhalb von 3 Monaten nach Erreichen eines klinischen Ansprechens auf die TSEBT verabreicht. Die Anschlusstherapie wurde bis zum Fortschreiten der Erkrankung oder dem Auftreten einer inakzeptablen Toxizität fortgeführt.^16^ Die Verabreichung einer vorab geplanten Folgebehandlung wurde dabei nicht als Ereignis im Sinne der Zeit bis zur nächsten Behandlung (TTNT) gewertet.^17^

Von September 2019 bis August 2024 wurden insgesamt 220 Patienten in neun Behandlungszentren mit primär kutanem Lymphom im S‐MISR‐Register rekrutiert. In unsere Studie wurden insgesamt 35 Patienten eingeschlossen, darunter 21 mit SS (10 mit *de novo* SS und 11 sekundär aus einer MF) sowie 14 mit eMF.

Die immunhistochemische CD30‐Expression war bei 27 Patienten verfügbar, wobei die Ergebnisse bei neun Patienten (33%) positiv waren, mit einer mittleren Expression von 10% (IQR 5–30). Alle Patienten hatten eine aktive oder progrediente Erkrankung nach früheren Behandlungen (Median 3 Behandlungen, Spanne 1–8). Der behandelnde Radioonkologe entschied auf der Grundlage der klinischen Gegebenheiten über die Dosis der Strahlentherapie. Keiner der Patienten in unserer Kohorte erhielt eine begleitende Therapie zur Bestrahlung. Die mittlere Bestrahlungsdosis betrug 12 Gy (Bereich: 8–12 Gy) in sechs verschiedenen Bestrahlungspositionen (Sechs‐Doppel‐Feld‐Technik). Aufgrund der COVID‐19‐Pandemie wurden 14 Patienten (40%) mit einem ultra‐hypofraktionierten Konzept behandelt.^18^ Einunddreißig (89%) Patienten erhielten zusätzlich lokale Elektronenfelder in den während der TSEBT abgeschirmten Bereichen. Gleichzeitig erhielten 19 Patienten (54%) eine lokale *Boost*‐Bestrahlung im Bereich der pathologisch vergrößerten axillären und inguinalen Lymphknoten mit einer medianen Dosis von 9 Gy (Bereich: 4–30). Zu den Ausgangsuntersuchungen gehörten die Anamnese und Fragebögen zur Lebensqualität. Die TSEBT‐Toxizitäten und die Lebensqualität wurden zunächst wöchentlich während der Bestrahlung, anschließend nach 6–8 Wochen, nach 12 Wochen und dann alle 3 Monate bis zum Rezidiv oder Progress der Erkrankung untersucht. Neunundzwanzig (83%) Patienten füllten die Fragebögen Skindex‐29 und EORTC QLQ‐C30 (Version 3.0) vor und innerhalb von 12 Wochen nach der TSEBT aus.

Darüber hinaus wurden von allen Patienten zu Beginn der Studie und im Rahmen der Nachuntersuchungen Blutproben zur weiterführenden Diagnostik entnommen. Die Blutproben wurden innerhalb von 4 Stunden nach der Entnahme im Labor auf folgende Parameter untersucht: Routine‐Blutwerte, peripheres Blutbild, β_2_‐Mikroglobulin, L‐Lactatdehydrogenase (LDH), C‐reaktives Protein (CRP), löslicher Interleukin‐2‐Rezeptor (sIL‐2R), lösliches Interleukin‐6 (sIL‐6), Neopterin, Tumornekrosefaktor (TNF)‐α sowie Immunglobulinkonzentrationen. Zusätzliche Blutproben (7 mL) wurden vor und nach der Strahlentherapie in Serumröhrchen entnommen, und die Proben wurden 10 min lang bei 2500 g (U/min) zentrifugiert. Die Serumfraktionen wurden in Kryoröhrchen (Greiner, Kremsmünster, Österreich) gefüllt und bei –80 °C gelagert, bevor sie mithilfe von ELISA‐Kits (Bio‐Techne, Wiesbaden, Deutschland) auf löslichen Programmierter Zelltod‐Ligand 1 (sPD‐L1, *soluble programmed death‐ligand 1*), lösliches CD30 (sCD30) und lösliches thymus‐ und aktivierungsreguliertes Chemokin (sTARC, *soluble thymus and activation‐regulated chemokine*) untersucht wurden.^38^

### Therapieansprechen

Für die Bewertung des Therapieansprechens wurden internationale Kriterien herangezogen.^19^ Zu den Respondern nach 3 Monaten gehörten Patienten mit komplettem Ansprechen (CR, *complete response*), sehr guter partieller Remission (VGPR, *very good partial remission*) und partieller Remission (PR, *partial remission*), während zu den Non‐Respondern Patienten mit stabiler Erkrankung (SD, *stable disease*) oder progressiver Erkrankung (PD, *progressive disease*) gezählt wurden. Der primäre Endpunkt war die Ansprechrate gemäß den internationalen Empfehlungen.^3^, ^17^, ^19^

Sekundäre Endpunkte waren die Zeit bis zur nächsten Behandlung (TTNT; der Zeitraum ab Beginn der TSEBT) und das progressionsfreie Überleben (PFS; der Zeitraum vom Beginn der TSEBT bis zum Fortschreiten der Erkrankung, Rückfall oder Tod). Explorative Ziele waren Daten zur Lebensqualität und Veränderungen von Blutparametern in Blutproben, die bei Studienbeginn und in der Nachbeobachtungsphase entnommen wurden, um die Veränderungen von Biomarkern zu bewerten.

### Statistische Analyse

Die statistischen Analysen wurden mit IBM SPSS Statistics Version 29.0 durchgeführt. Die Ergebnisse werden sowohl als absolute Zahlen als auch als relative Werte dargestellt. Wir verwendeten Chi‐Quadrat‐Tests und den exakten Test nach Fisher, um die Beziehungen zwischen kategorischen Variablen zu untersuchen. Kontinuierliche Variablen werden durch ihre Mediane, Interquartilsbereiche (IQR) und Quartile beschrieben. Mithilfe von Kaplan‐Meier‐Kurven wurde der Median des PFS und der TTNT ermittelt, wobei die entsprechenden Konfidenzintervalle (KI) durch Standardannäherungen bestimmt wurden.^20^ Der Log‐Rank‐Test wurde verwendet, um die Zeit bis zum Auftreten von Endpunkten zwischen verschiedenen Gruppen zu vergleichen. Die *Hazard Ratio* aus dem *Cox Proportional Hazards Model* und die entsprechenden KI stellen ein zusätzliches Maß für die Effektgröße dar. Die Unterschiede der Biomarker vor und nach der Behandlung wurden anhand der Medianwerte dargestellt; die p‐Werte wurden mithilfe des Wilcoxon‐Signed‐Rank‐Tests berechnet. Die Korrelation zwischen den Baseline‐Schätzungen und den klinischen Parametern wurde anhand der Spearman‐Rank‐Korrelation bewertet. Für den Vergleich der modifizierten *Severity‐Weighted Assessment Tool* (mSWAT)‐ und Skindex‐29‐(Sub‐) Scores vor und nach TSEBT wurde eine Bonferroni‐Korrektur vorgenommen. Korrigierte p‐Werte < 0,05 wurden als signifikant angesehen.

## ERGEBNISSE

Die Patientenmerkmale sowie weitere klinische und biochemische Parameter sind in Tabelle 1 dargestellt. Das mediane Alter der gesamten Kohorte betrug 68 Jahre (IQR 60–76). Die mediane Nachbeobachtungszeit betrug 9 Monate (IQR 4–24). Die Patienten zeigten innerhalb von 6 Wochen nach Beginn der TSEBT eine deutliche Verbesserung des mSWAT‐Wertes (p < 0,001). Der mediane mSWAT‐Score vor der Bestrahlung betrug 99 (IQR 80–100) gegenüber 20 (IQR 6–41) in der 6. Woche und 12 (IQR 5–29) im 3. Monat nach TSEBT. Hinsichtlich der Lymphadenopathie lag die Ansprechrate in der gesamten Kohorte bei 89%, wobei die Ansprechrate bei Patienten, die eine lokale Bestrahlung der Lymphknoten erhielten, bei 100% lag, verglichen mit Patienten, die keine Bestrahlung der Lymphknoten erhielten (57%, p = 0,013). Zur Analyse der Blutbeteiligung wurden nur bei 20 Patienten gepaarte Bluttests durchgeführt, hier lag eine Ansprechrate von 80% vor (40% CR der aberranten T‐Zell‐Populationen nach TSEBT und 40% hatten ein teilweises Ansprechen), während bei 20% die aberranten T‐Zell‐Populationen nach TSEBT stabil blieben.

**TABELLE 1:** Patienten‐, Krankheits‐ und Behandlungsmerkmale.

Merkmal	Erythrodermatische MF, n = 14 (%)	Sézary‐Syndrom, n = 21 (%)	p‐Wert
Alter (median), Jahre (IQR)	64 (17)	69 (13)	0,18
Geschlecht			
Mann	11 (79)	16 (76)	1,0
Frau	3 (21)	5 (24)	
Baseline Performance Status (ECOG)			
I	8 (57)	11 (52)	1,0
II	6 (43)	10 (48)	
Baseline mSWAT score, median (IQR)	91 (22)	100 (20)	0,78
Klinische Symptome zum Zeitpunkt der Überweisung			1,0
Juckreiz	14 (100)	21 (100)	
Hyperkeratose	10 (71)	15 (71)	
Alopezie	10 (71)	14 (67)	
Juckreiz‐Skala vor TSEBT (IQR)	8 (2)	8 (4)	0,49
LDH erhöht	8 (57)	16 (76)	0,42
Leukozyten erhöht	3 (21)	9 (45)	0,28
Lymphknoten Beteiligung	9 (64)	17 (81)	0,42
Großzellige Transformation vor der Bestrahlung	3/12 (25)	2/15 (13)	0,63
Anzahl der vorangegangenen Therapien (Bereich)	3 (1‐6)	4 (2‐8)	0,12
Mediane Zeit von der Diagnose bis zur TSEBT, Monate (IQR)	35 (13)	28 (54)	0,96
Angewandte TSEBT‐Dosierungsschemata			1,0
8 Gy in 2 oder 4 Fraktionen	6 (43)	8 (38)	
12 Gy in 8 Fraktionen	8 (57)	13 (62)	
Antibiotikatherapie gleichzeitig mit TSEBT	4 (29)	7 (33)	1,0
Zweite TSEBT‐Behandlung	2 (14)	5 (24)	0,67
Lymphknoten Boost‐Bestrahlung	6 (43)	13 (62)	0,32
Ansprechrate nach TSEBT			0,61
CR	1 (7)	1 (5)	
VGPR	8 (57)	7 (33)	
PR	4 (29)	10 (48)	
Erhaltungstherapie or anschießende Therapie bei Respondern			0,69
Ja	9 (69)	14 (78)	
Nein	4 (31)	4 (22)	

*Abk*.: CR, komplettes Ansprechen; ECOG, *Eastern Cooperative Oncology Group*; IQR, Interquartilsabstand; LDH, Laktatdehydrogenase; MF, Mycosis fungoides; mSWAT, modifiziertes *Severity Weighted Assessment Tool*; PR, partielles Ansprechen; SS, Sézary‐Syndrom; TSEBT, Ganzhautelektronenbestrahlung; VGPR, sehr gutes partielles Ansprechen

Die Gesamtansprechrate (*overall response rate*, ORR) nach 3 Monaten betrug 89%, mit 14 PR (40%), 15 VGPR (43%) und 2 CR (6%). Es gab keinen signifikanten Unterschied in der ORR zwischen eMF und SS (93% bzw. 86%). Im Vergleich dazu lag die SD‐Rate in der eMF‐Gruppe bei 7% und in der SS‐Gruppe bei 9%. Nur bei einem Patienten in der SS‐Kohorte kam es direkt nach der TSEBT zu einer Progression. Fünfundzwanzig (71%) Patienten erhielten nach der TSEBT eine Folgebehandlung. Beispiele für klinische Hautreaktionen sind in den Abbildungen S1 und S2 (Online‐Supplement) zu sehen.

In der Gesamtkohorte betrug die mediane TTNT 20 Monate (95%‐KI: 10–30) und das mediane PFS 14 Monate (95%‐KI: 4–24). Es zeigten sich keine signifikanten Unterschiede bei den Ansprechraten, der TTNT und dem PFS hinsichtlich der verwendeten Strahlendosis und der Fraktionierung. Sieben Patienten erhielten eine zweite TSEBT‐Bestrahlung nach einem medianen Abstand von 13 Monaten. Nach der Bestrahlung betrug das 5‐Jahres‐Gesamtüberleben 92% bei den eMF‐Patienten und 36% bei den SS‐Patienten.

### Anschließende systemische Behandlungen

Bei 31 Respondern wurde der Nutzen einer nachfolgenden Therapie analysiert. Die am häufigsten verabreichte Folgetherapie war Mogamulizumab (n = 9), gefolgt von oralen Retinoiden (n = 5), Methotrexat (n = 5), pegyliertem Interferon‐α (n = 3), extrakorporaler Photopherese (n = 2) sowie weiteren Therapien (n = 7). Die mediane Nachbeobachtungszeit war zwischen der Gruppe mit anschließender Therapie und der Gruppe ohne anschließende Therapie ähnlich. Bei den Patienten, die eine anschließende Therapie erhielten, betrug die mediane TTNT 30 Monate (95%‐KI: 14–46, p = 0,001). Im Gegensatz dazu lag die mediane TTNT bei den Respondern ohne anschließende Therapie bei nur 6 Monaten (95%‐KI: 2–10). Darüber hinaus betrug das mediane PFS der Patienten in der Gruppe mit anschließender Therapie 23 Monate (95%‐KI: 9–37), während es bei den Patienten ohne anschließende Therapie nur 5 Monate betrug (95%‐KI: 2–8; p < 0,001). In der Subgruppenanalyse konnten die Ergebnisse für die eMF‐Kohorte (42 vs. 13 Monate, p = 0,036) und die SS‐Kohorte (20 vs. 3 Monate, p <0,001) bestätigt werden. Hinsichtlich der Wahl der anschließenden Therapieform konnten wir keinen signifikanten Unterschied feststellen, was wahrscheinlich auf die geringe Stichprobengröße für die Subgruppenanalyse zurückzuführen ist.

### Toxizitätsprofil

Bei 33 (94%) Patienten traten akute Toxizitäten vom Grad 1 und bei 17 (49%) Toxizitäten vom Grad 2 auf. Die häufigsten Toxizitäten ab Grad 2 betrafen kutane Reaktionen (n = 17), Alopezie (n = 6) und Nagelverlust (n = 2). Zwei (6%) Patienten mit Sézary‐Syndrom entwickelten eine Toxizität vom Grad 3 (Bakteriämie) und wurden während der Strahlentherapie antibiotisch behandelt. Es zeigten sich keine signifikanten Unterschiede in den strahlenbedingten Toxizitätsgraden in Abhängigkeit von applizierter Gesamtdosis, Fraktionierung oder Erkrankungstyp. Im Rahmen der nachfolgenden Therapien wurden keine behandlungsassoziierten Toxizitäten ≥ Grad 3 beobachtet.

### Gesundheitsbezogene Lebensqualität (*health‐related quality of life*, HRQoL)

Hinsichtlich des Juckreizes, des Skindex‐29 und der EORTC‐QLQ‐C30‐Subskalen vor der Behandlung konnten wir keinen signifikanten Unterschied zwischen eMF‐ und SS‐Patienten feststellen. Auf der Grundlage des Skindex‐29‐Scores wurde innerhalb von 4 Wochen nach Beginn der TSEBT‐Behandlung eine signifikante Verbesserung in allen Subdomänen festgestellt (Tabelle 2). Unter 28 Patienten (80%) mit Juckreiz trat bei mehr als zwei Dritteln (71%) nach der TSEBT eine Verbesserung ein und bei 25% war der Juckreiz vollständig verschwunden.

**TABELLE 2:** Skindex‐29, EORTC‐QLQ‐C30, Juckreiz und mSWAT im Median (Interquartilsbereich) vor und nach der Bestrahlung (n = 35).

	Skindex29 global score	Symptome	Emotionen	Funktionen	Frage 18	EORTC QLQ‐C30 global score	Juckreiz‐Skala	mSWAT
Baseline	112 (37)	26 (9)	38 (14)	42 (21)	3,5 (1)	6 (5)	8 (2)	100 (20)
3 Monate nach Radiotherapie	79 (63)	18 (12)	25 (21)	29 (27)	2 (2)	7 (6)	2 (5)	14 (25)
Original p‐Werte^*^	0,0008	0,00002	0,00004	0,0004	0,002	0,007	0,000005	0,00003
Bonferroni‐korrigierte p‐Werte^**^	0,0064	0,00016	0,00032	0,0032	0,016	0,056	0,00004	0,00024

Abk.: EORTC QLQ‐C30, European Organisation for Research and Treatment of Cancer Quality of Life Questionnaire‐Core 30; mSWAT, modifiziertes Severity Weighted Assessment Tool; Q18, Item 18 des EORTC QLQ‐C30 zur Erfassung der Juckreizintensität; Skindex‐29, 29 Items umfassender dermatologiespezifischer Lebensqualitätsfragebogen

* Signed‐rank test

** Es wurden acht Vergleiche durchgeführt.

### Explorative Analyse potenzieller Biomarker

Die Untersuchung von blutbasierten Biomarkern ermöglicht Auskunft über die Prognose verschiedener hämatologischer Malignome. Daher wurden vorliegend zusätzlich die hämatologischen und serologischen Blut‐Veränderungen bei eMF/SS analysiert. Unser Ziel war es, hämatologische und serologische Veränderungen im Zusammenhang mit dem klinischen Erscheinungsbild zu bewerten und diese mit den klinischen Ergebnissen zu korrelieren.

Bei 33 Patienten (94%; 13 mit eMF und 20 mit SS) wurden zu Studienbeginn und bei der ersten Nachuntersuchung (6–12 Wochen nach TSEBT) paarweise Blutproben entnommen. Bei sieben Patienten lagen Paare von Blutproben zu Beginn und bei Krankheitsprogression vor. Zunächst wurden die Konzentrationen potenzieller Biomarker in den Serumproben von Patienten mit eMF und SS zu Studienbeginn bestimmt. Patienten mit SS zeigten dabei infolge der Tumorzellbeteiligung im peripheren Blut eine erhöhte Lymphozytenzahl (Median 4185 vs. 1060 Zellen/µl; p = 0,02), höhere CD4^+^‐Lymphozytenwerte (Median 2170 vs. 575 Zellen/µl; p = 0,04) sowie eine Tendenz zu einer erhöhten CD3^+^‐Zellzahl (Median 1765 vs. 784 Zellen/µl; p = 0,065). Kein relevanter Unterschied zeigte sich im CD4:CD8‐Verhältnis (Median 4,5 vs. 3,2; p = 0,24).

Die sPD‐L1‐Ausgangswerte korrelierten signifikant mit der kutanen Krankheitslast (r = 0,608; p = 0,047). Interessanterweise waren sowohl die sIL‐2R‐Werte (Median 3279 vs. 833 pg/ml; p = 0,036) als auch die LDH‐Spiegel (Median 308 vs. 234 pg/ml; p = 0,057) in der SS‐Kohorte im Vergleich zur eMF‐Gruppe deutlich erhöht. Alle übrigen Biomarker zeigten in beiden Kohorten vergleichbare Konzentrationen.

Nach der TSEBT‐Behandlung sank die Leukozytenzahl in der gesamten Studienkohorte signifikant (p < 0,001). Darüber hinaus konnte bei SS‐Patienten zwischen dem Ausgangs‐ und dem Vergleichswert im Rahmen der Nachuntersuchung eine signifikante Reduktion des Medians bei folgenden Biomarker beobachtet werden: LDH (p = 0,005), Sézary‐Zellen (p = 0,003), CD3 (p = 0,006), CD4 (p = 0,003), CD19 (p = 0,05), sIL‐2R (p = 0,015) und sCD30 (p = 0,04). Patienten mit eMF zeigten hier keine signifikante Veränderung zwischen dem Ausgangswert und der Nachuntersuchung (p > 0,05). Allerdings ergab sich bei Patienten mit eMF eine signifikante Reduktion von β_2_‐Mikroglobulin (p = 0,03) und IgG (p = 0,05) zwischen Ausgangswert und Nachuntersuchung. Die LDH‐ (p = 0,019) und sTARC‐Werte (p = 0,043) stiegen bei Krankheitsprogression oder Rezidiv signifikant an.

## DISKUSSION

In der vorliegenden Studie wurde die Wirksamkeit einer niedrig dosierten TSEBT bei Patienten mit eMF und SS untersucht. Darüber hinaus wurden verschiedene explorative Parameter zu Beginn und nach Abschluss der Behandlung ausgewertet. Aus unserer Analyse ergaben sich folgende Erkenntnisse: Erstens war die niedrig dosierte TSEBT mit einer hohen objektiven Ansprechrate (ORR) und einer raschen Verbesserung der Lebensqualität (QoL‐Scores) assoziiert. Zweitens könnte eine nachfolgende systemische Therapie nach niedrig dosierter TSEBT bei eMF/SS mit einem klinischen Nutzen verbunden sein. Drittens wurden verschiedene potenzielle Biomarker im peripheren Blut identifiziert, deren weiterer Einsatz zur Auswahl einer optimalen systemischen Anschlusstherapie prospektiv untersucht werden sollte.

Die Auswertung der Lebensqualitätsdaten anhand des Skindex‐29 ergab, dass Risikogruppen – darunter Frauen sowie Patienten mit erhöhtem LDH‐Wert, Alopezie oder hohem mSWAT‐Wert – schlechtere Ergebnisse aufwiesen als andere Patienten mit MF.^6^ Niedrig dosierte TSEBT ist in der Regel mit geringeren Toxizitäten in fortgeschrittenen Fällen verbunden.^21^, ^22^ Darüber hinaus verbessert sich die Lebensqualität innerhalb von 4–8 Wochen nach der Bestrahlung deutlich.^9^ Außerdem soll TSEBT die Tumorlast im peripheren Blut verringern.^11^, ^12^ In einer Subgruppenanalyse der SS‐Kohorte zeigte sich zwischen Ausgangs‐ und Follow‐up‐Wert eine signifikante Reduktion der Medianwerte für die Biomarker LDH, Leukozytenzahl, Sézary‐Zellen, CD3⁺‐ und CD4⁺‐T‐Zell‐Lymphozyten, CD19⁺‐B‐Zell‐Lymphozyten, sIL‐2R sowie sCD30⁺‐T‐Zell‐Lymphozyten. Derzeit werden in weiteren prospektiven Studien potenzielle lösliche Biomarker im Blutserum der Patienten vor und nach der Behandlung untersucht.^15^, ^23^

Zielgerichtete Therapien bei MF und SS richten sich gegen verschiedene Oberflächenmoleküle, die auf Tumorzellen exprimiert werden. Kürzlich wurde der Anti‐CCR4‐Antikörper Mogamulizumab für Patienten mit fortgeschrittener MF oder SS zugelassen.^24^, ^25^ Die MAVORIC‐Studie zeigte, dass Mogamulizumab die TTNT bei Patienten mit SS um bis zu 20 Monate gegenüber 12 Monaten bei B1 und nur 7 Monaten bei B0 verbessert. Die ORR betrug 46% bei SS gegenüber 26% bei B1 und 16% bei B0.^26^

Die Ansprechrate der Blutlast war jedoch höher als die der kutanen Manifestationen (68% vs. 42%), was vermutlich auf natürliche Killerzellen zurückzuführen ist, die für die antikörperabhängige zellvermittelte Zytotoxizität von Mogamulizumab essenziell sind.^26^

Mögliche Kombinationen zwischen Mogamulizumab und TSEBT wurden vorgeschlagen und könnten sinnvoll sein.^27^, ^28^, ^29^ In unserer Analyse lag die ORR für eMF‐Patienten bei 93% und die TTNT bei 42 Monaten, verglichen mit 86% ORR und 20 Monaten TTNT bei SS‐Patienten, die nach der TSEBT eine Folgetherapie erhielten. Die Anzahl natürlicher Killerzellen nahm nach TSEBT nicht ab, was die Wirksamkeit von Mogamulizumab als anschließende Therapieoption begünstigen könnte.^30^ Kürzlich empfahlen Assaf et al. die Kombination von Mogamulizumab mit einer Strahlentherapie von Beginn an, um die Ergebnisse zu verbessern.^31^ In einer multizentrischen retrospektiven Studie wurde mit pegyliertem Interferon alfa eine 38%ige PR bei MF im Stadium IIIA–B (n = 8) mit 8 Monaten TTNT erzielt.^32^

Im Stadium IVA1 (n = 18) lag die ORR bei vergleichsweise hohen 56% und war mit einem medianen TTNT von 11 Monaten assoziiert. Bei sieben Patienten im Stadium IVA2 lag die ORR bei 57% und eine TTNT von 14 Monaten. Zwei Drittel der Fälle erhielten eine Kombinationstherapie (am häufigsten mit ECP oder Bexaroten), die einer Monotherapie überlegen war.^32^ Die Rate der Toxizitäten ≥ Grad 3 lag zwischen 22% und 29%.^32^, ^33^ Ähnlich wie unsere Daten empfehlen auch die kürzlich veröffentlichten EORTC‐Konsensus‐Leitlinien Interferone, orale Retinoide, Methotrexat und extrakorporale Photopherese als Erhaltungstherapien für die Behandlung von MF/SS.^8^ Mögliche Wirkmechanismen der TSEBT bei SS beruhen auf der Exposition zirkulierender Tumorzellen gegenüber niedrig dosierter Strahlung beim Passieren oberflächlicher Gefäße. Dies kann zur Aktivierung des Transkriptionsfaktors NF‐κB führen, der an der Induktion und Regulation relevanter Gene und Zytokine beteiligt ist.^34^

Darüber hinaus führt die Strahlentherapie zu verschiedenen genetischen Veränderungen in den Tumorzellen, die das Immunsystem betreffen, entzündungsfördernde Signale erzeugen, die Mikroumgebung des Tumors verändern und tumorinfiltrierende Lymphozyten (TIL) rekrutieren.^35^ Des Weiteren rekrutieren Tumorzellen Chemokine und TIL im Bestrahlungsfeld. Zusätzlich werden reaktive Sauerstoffspezies (*reactive oxygen species*, ROS) gebildet, Kinasen werden durch DNA‐Schäden aktiviert und natürliche Killerzellen bilden und präsentieren aktivierende Liganden, was zur Rekrutierung dendritischer Zellen führt. Diese Phänomene unterdrücken die Tumorzellen in der nahen Mikroumgebung und beeinflussen das Wachstum von bestrahlten und nicht bestrahlten Läsionen.^35^, ^36^ Daher könnten zusätzliche immunmodulierende Behandlungen oder Immuntherapien die lokalen und systemischen Antitumor‐Immunreaktionen nach einer Strahlentherapie verbessern.^37^

Unsere Studie weist mehrere Limitationen auf, darunter insbesondere die geringe Fallzahl. Dabei ist jedoch die Seltenheit der Erkrankung sowie der gewählte Erhebungszeitraum zu berücksichtigen. Außerdem handelt es sich um eine eher heterogen verlaufende Krankheit, sodass Studien mit homogenen Gruppen für aussagekräftige klinische Empfehlungen schwierig durchzuführen sind. Darüber hinaus entschied der behandelnde Dermatologe in einzelnen Fällen, je nach Krankheitsstadium und Komorbiditäten, über die weitere Behandlung, was teilweise zu unterschiedlichen Empfehlungen führte. Aufgrund der COVID‐19‐Pandemie während unserer Studie standen nur in 86% der Fälle gepaarte Bluttests für den Vergleich löslicher Biomarker zur Verfügung und 40% der Patienten erhielten ultra‐hypofraktionierte Therapien. In dieser Studie sind mehr als die Hälfte der SS‐Patienten sekundär in eine MF übergegangen, was ungewöhnlich hoch ist und wahrscheinlich auf die kleine und heterogene Kohorte zurückzuführen ist. Trotz dieser Einschränkungen unterstützen unsere Ergebnisse die Durchführbarkeit einer niedrig dosierten TSEBT in Kombination mit einer anschließenden Therapie bei Patienten mit eMF und SS. Die Ergebnisse könnten als Grundlage für zukünftige Studien dienen.

Es ist wichtig zu beachten, dass die erreichte Kontrolle durch TSEBT oft nur vorübergehend ist und das Risiko eines Rückfalls relativ hoch ist. Daher wird empfohlen, eine Folge‐ oder Erhaltungstherapie in Erwägung zu ziehen. Die anschließende Therapieform kann mit Hilfe von Biomarkern, die potenzielle Rückschlüsse auf die Aktivierung des Immunsystems ermöglichen, überprüft werden. Zukünftig ist es wichtig, dass weitere prospektive Studien folgen, um die Aussagekraft der Ergebnisse zu erhöhen. Demnach sind weitere randomisierte Studien erforderlich, um die Wirksamkeit von niedrig dosierter TSEBT in Kombination mit systemischen Therapien bei Patienten mit eMF und SS zu validieren.

### Schlussfolgerungen

In der vorliegenden Studie konnte gezeigt werden, dass eine niedrig dosierte TSEBT die Krankheit unter Kontrolle hält und in Zukunft vermehrt Bestrahlungen möglich sind. Die Lebensqualität der Patienten wird durch die Bestrahlung deutlich verbessert und die Toxizität ist relativ gering. Wir empfehlen eine anschließende systemische Therapie, da Biomarker auf eine Reaktion des Immunsystems hinweisen und Patienten, die eine systemische Therapie erhalten, möglicherweise davon profitieren.

## DANKSAGUNG

Wir danken den teilnehmenden Patienten und ihren Familien, Krankenschwestern, Ärzten, Physikern und Strahlentherapeuten für ihre Beiträge und Unterstützung. Ebenfalls sind wir Frau Dr. Julia Siats, Herrn Prof. Carsten Weishaupt und Prof. Burkhard Greve für die Bereitstellung der erforderlichen Daten sehr dankbar. Wir danken Dr. Julia Siats für die sprachliche Überarbeitung des Manuskripts.

Open access Veröffentlichung ermöglicht und organisiert durch Projekt DEAL.

## FINANZIERUNG

Die Forschung wurde vom Fond für Innovative Medizinische Forschung der Medizinischen Fakultät der Universität Münster gefördert (Fördernummer: EL112102).

## INTERESSENKONFLIKT

K. E. erhielt Beratungs‐ und Vortragshonorare von Kyowa Kirin und Gilead Sciences. J. P. N. erhielt Mittel für Reisen und Kongressteilnahmen von TEVA und Novartis sowie Beratungshonorare von TEVA, Almirall, Biogen, Novartis, Kyowa Kirin, Innate Pharma, Takeda, Actelion, UCB Pharma und Recordati. S. M. erhielt Honorare und Reisekostenzuschüsse von Kyowa Kirin. R. S. erhielt Honorare von Kyowa Kirin, 4SC AG, Stemline, Recordati und Innate Pharma. E. L. erhielt Honorare von Takeda. C. P. erhielt Honorare von MSD, BMS, Novartis, AbbVie, Sanofi, Merck, Serono, Sun Pharma, Pierre Fabre, LEO und Alery Therapeutics. Die übrigen Autoren erklären, dass keine Interessenkonflikte bestehen.

Der Abstract wurde als mündliche Präsentation angenommen und auf der Jahrestagung 2024 der EORTC Cutaneous Lymphoma Tumour Group vorgestellt, die vom 9. bis 11. Oktober 2024 in Lausanne, Schweiz, stattfand. Der Abstract wurde im European Journal of Cancer (EJC) veröffentlicht.

Die übrigen Autoren erklären, dass keine Interessenkonflikte bestehen.

## Supporting information



Supplementary information

Supplementary information
