# Supplementary figures and images for: Niedrig dosierte Ganzhaut‐Elektronenbestrahlung bei erythrodermatischer Mycosis fungoides und Sézary‐Syndrom: Ergebnisse aus der prospektiven S‐MISR‐Studie
Source: J Dtsch Dermatol Ges. 2025 Dec 11;23(12):1534–43. [Article in German] doi: 10.1111/ddg.15851_g (PMC12697331; doi:10.1111/ddg.15851_g)

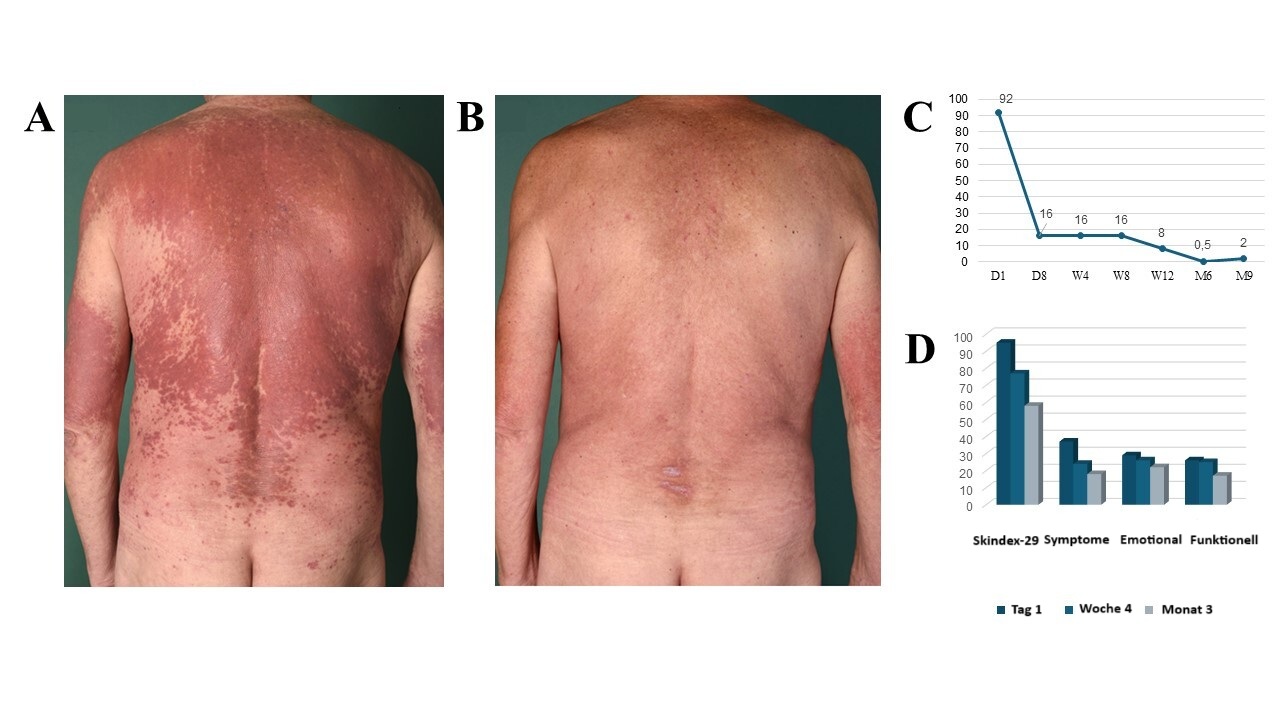

Supplement: Supplementary file 1 — Supplementary information [file DDG-23-1534-s002.jpg]

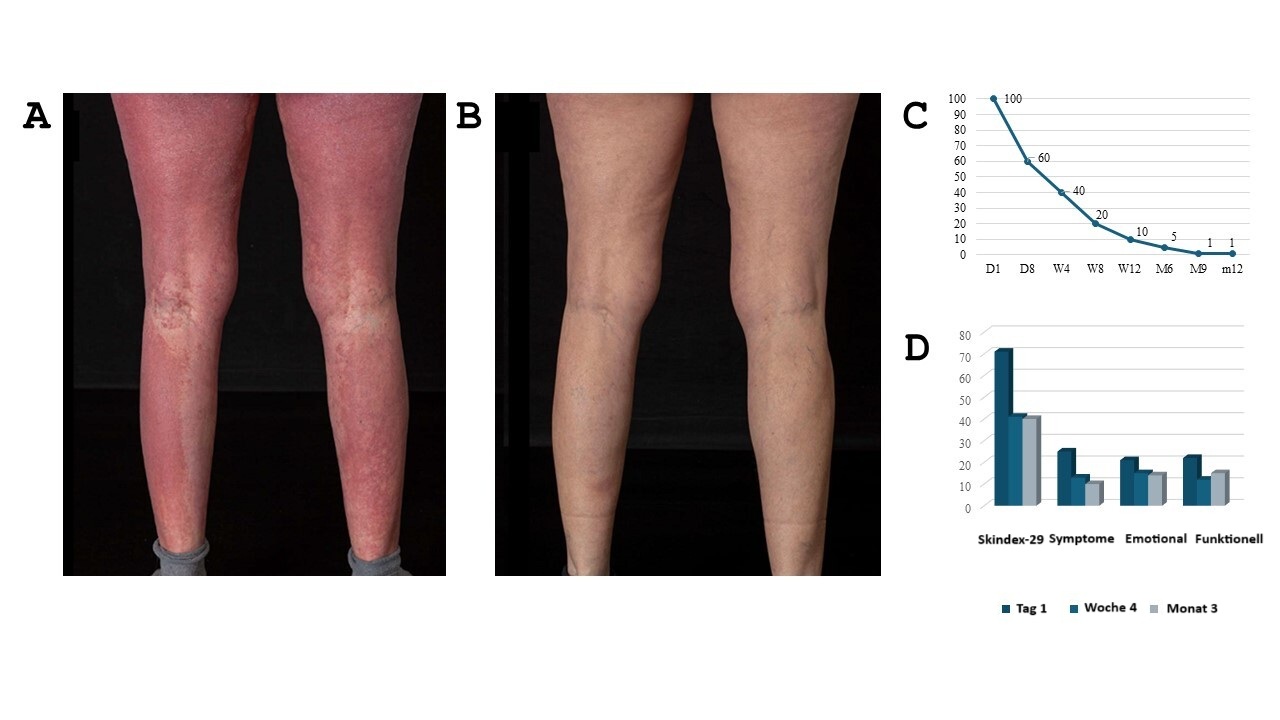

Supplement: Supplementary file 2 — Supplementary information [file DDG-23-1534-s001.jpg]
